# Expression of the Receptor for Advanced Glycation End Products in Epicardial Fat: Link with Tissue Thickness and Local Insulin Resistance in Coronary Artery Disease

**DOI:** 10.1155/2016/2327341

**Published:** 2015-12-14

**Authors:** Elena Dozio, Elena Vianello, Silvia Briganti, John Lamont, Lorenza Tacchini, Gerd Schmitz, Massimiliano Marco Corsi Romanelli

**Affiliations:** ^1^Department of Biomedical Sciences for Health, University of Milan, Via L. Mangiagalli 31, 20133 Milan, Italy; ^2^Diabetology and Metabolic Disease Unit, I.R.C.C.S. Policlinico San Donato, Piazza E. Malan 1, 20097 San Donato Milanese, Italy; ^3^Randox Laboratories Ltd., R&D, 55 Diamond Road, Crumlin, Antrim, Belfast BT29 4QY, UK; ^4^Institute for Clinical Chemistry and Laboratory Medicine, University of Regensburg, Universitätsstraße 31, 93053 Regensburg, Germany; ^5^Service of Laboratory Medicine 1-Clinical Pathology, I.R.C.C.S. Policlinico San Donato, Piazza E. Malan 1, 20097 San Donato Milanese, Italy

## Abstract

Increased expression of receptor for advanced glycation end products (RAGE) in adipose tissue has been associated with inflammation, adipocyte hypertrophy, and impaired insulin signal. Epicardial adipose tissue (EAT), a visceral fat surrounding the myocardium, is potentially involved in the onset/progression of coronary artery disease (CAD). To date, the role of RAGE in EAT has not been explored much. We examined whether the RAGE expression in EAT was associated with EAT adiposity and metabolic dysfunctions normally found in CAD patients. EAT samples were obtained from 33 patients undergoing open-heart surgery. EAT expression of RAGE, GLUT4, adiponenctin, GLO1, HMGB1, TLR-4, and MyD88 was analyzed by microarray. EAT thickness was quantified by echocardiography. Anthropometric measures and clinical parameters were taken. BMI, HOMA-IR, and LAP indices were calculated. With increasing RAGE expression in EAT we observed increases in EAT thickness, reduced expression of GLUT4, adiponectin, and GLO1, and elevations of HMGB1, TLR-4, and MyD88. There were significant correlations between RAGE and EAT thickness and between RAGE and the genes. LAP was higher in patients with increased RAGE expression. Our data suggest that in CAD patients RAGE may be involved in promoting EAT adiposity and metabolic dysfunction, such as impaired insulin signaling.

## 1. Introduction

The role of epicardial adipose tissue (EAT) in the onset and progression of coronary artery disease (CAD) is recognized [1], but the mechanisms and mediators promoting and linking EAT dysfunctions and CAD still need to be understood and described better.

The receptor for advanced glycation end products (RAGE) is a multiligand receptor that binds advanced glycation end products (AGE) and other endogenous nonglycated peptides, such as ligand mobility group box 1 (HMGB1), many of which are important regulators of the inflammatory process [2]. Although RAGE was initially implicated in cardiovascular complications related to diabetes [3, 4], recent reports have suggested its central role in inflammation and inflammation-associated dysfunctions, such as obesity, metabolic syndrome, and atherosclerosis, even in nondiabetic conditions [5–8].

In CAD, EAT displays inflammatory features due to infiltrated macrophages and T cells and reduced production of protective factors in favor of detrimental proinflammatory mediators. This inflammatory condition may in turn contribute to the progression of atherosclerosis besides exacerbating metabolic complications in EAT [9, 10].

On the basis of the potential role of RAGE in adipogenesis, inflammation, and insulin resistance [5–8], we explored whether its expression in EAT was associated with EAT adiposity and the metabolic dysfunctions, such as impaired insulin signaling, normally found in CAD patients.

## 2. Materials and Methods

### 2.1. Study Population

Thirty-three male CAD patients undergoing coronary artery bypass grafting (CABG) surgery were enrolled in the study during their hospitalization. Exclusion criteria were acute myocardial infarction within the last month, previous or current malignant disease, major abdominal surgery within the previous 6 months, renal and liver diseases, end-stage heart failure, and more than 3% change in body weight in the previous three months. Anthropometric measures were recorded. Body mass index (BMI) was calculated by dividing the weight (in Kg) by the square of the height (in meters). The study protocol, conducted in accordance with the Declaration of Helsinki, as revised in 2013, was approved by the local ethics committee (ASL Milano Due, Protocol 2516). Patients gave their written informed consent to the protocol.

### 2.2. Blood Collection

Blood samples were collected after overnight fasting into pyrogen-free tubes with ethylenediaminetetraacetic acid as anticoagulant. Fasting glucose, glycated hemoglobin, insulin, total and HDL cholesterol, triglycerides, and C-reactive protein (CRP) were quantified with commercial kits using Cobas 6000 analyzer (Roche Diagnostics, Milan, Italy), as previously reported [11, 12]. Precision was determined by the manufacturer using human samples and controls in an internal protocol with repeatability (*n* = 21) and intermediate precision (3 aliquots per run, 1 run per day, 21 days). Results for repeatability were glucose, 1% at 98.8 mg/dL and 0.9% at 245 mg/dL; HbA1c, 1.3% at 5.3% and 1.1% at 9.9%; insulin, 1.9% at 6.36 *μ*U/mL and 1.9% at 20.9 *μ*U/mL; total cholesterol, 1.1% at 88.5 mg/dL and 0.9% at 183 mg/dL; HDL, 0.4% at 53.4 mg/dL and 1% at 34.4 mg/dL; triglycerides, 0.9% at 125 mg/dL and 0.8% at 212 mg/dL; and CRP, 1.2% at 3.35 mg/L and 1.3% at 44.4 mg/L.

Results for intermediate precision were glucose, 1.3% at 96.9 mg/dL and 1.1% at 241 mg/dL; HbA1c, 1.4% at 5.3% and 1.5% at 9.9%; insulin, 2.6% at 6.36 *μ*U/mL and 2.8% at 20.9 *μ*U/mL; total cholesterol, 1.6% at 89.3 mg/dL and 1.6% at 188 mg/dL; HDL, 0.9% at 51.8 mg/dL and 1.5% at 34 mg/dL; triglycerides, 2% at 123 mg/dL and 1.6% at 206 mg/dL; and CRP, 2.9% at 29.1 mg/L and 1.9% at 43.6 mg/L.

LDL cholesterol was calculated with the Friedewald formula. Insulin resistance index (HOMA-IR) was calculated as follows: HOMA-IR = fasting insulin [*μ*U/mL] × fasting glucose [mmol/L]/22.5. The formula used for the lipid accumulation product (LAP) was (waist circumference [WC, cm] − 65) × (triglycerides [TG, mmol/L]).

### 2.3. Quantification of EAT

EAT quantification by echocardiography was performed in addition to the routine clinical examinations just before CABG surgery, usually one or two days before. Patients were examined by echocardiography using an M-mode color-Doppler VSF (Vingmed-System Five; General Electric, Horten, Norway) with a 2.5–3.5 MHz transducer probe. EAT thickness was measured as previously reported [13].

### 2.4. EAT Collection

EAT biopsy samples were harvested adjacent to the proximal right coronary artery prior to starting cardiopulmonary bypass pumping. Samples were stored in Allprotect Tissue Reagent (Qiagen, Hilden, Germany) at −20°C until RNA extraction.

### 2.5. RNA Extraction and Gene Expression Analysis

Total RNA was extracted from tissue with the RNeasy Lipid Tissue Kit according to the manufacturer's procedures (Qiagen). RNA concentration was quantified by NanoDrop 2000 (Thermo Scientific, Wilmington, Germany) and RNA integrity was assessed using the Agilent RNA 6000 Nano Kit and the Agilent 2100 Bioanalyzer (Agilent Technologies, Santa Clara, CA). Gene expression was analyzed with a one-color microarray platform (Agilent): 50 ng of total RNA was labeled with Cy3 using the Agilent Low Input Quick-Amp Labeling Kit-1 color, according to the manufacturer's directions. cRNA was purified with the RNeasy Mini Kit (Qiagen) and the amount and labeling efficiency were measured with NanoDrop. Hybridization was done using the Agilent Gene Expression Hybridization Kit and scanning with the Agilent G2565CA Microarray Scanner System.

Data were processed using Agilent Feature Extraction Software (10.7) with the single-color gene expression protocol and raw data were analyzed with ChipInspector Software (Genomatix, Munich, Germany). In brief, raw data were normalized on a single-probe level based on the array mean intensities and statistics were calculated using the SAM algorithm by Tusher et al. [14]. Changes were determined from normalized data.

### 2.6. Statistical Analysis

Data are expressed as mean ± SD or number and percentage. The normality of data distribution was assessed by the Kolmogorov-Smirnoff test. Quantitative variables were compared using Student's unpaired *t*-test and Mann-Whitney and Kruskal-Wallis tests, as appropriate. The *χ* ^2^ test was used for categorical variables. Relations between parameters were examined by the Spearman correlation test. Data were analyzed using GraphPad Prism 5.0 biochemical statistical package (GraphPad Software, San Diego, CA). A *p* value < 0.05 was considered significant.

## 3. Results

### 3.1. Patients

Demographic, anthropometric, and clinical characteristics of the patients are shown in Table 1. Patients were classified into two groups (Q1 and Q2) according to the median value of RAGE expression in EAT (168.33 arbitrary unit, A.U.). Patients were 17 in Q1 and 16 in Q2 and the mean value of RAGE expression was 140.29 A.U. in Q1 and 233.33 A.U. in Q2 (*p* < 0.001). No statistically significant differences were observed in clinical parameters between the two groups. There were higher percentages of dyslipidemic patients and more patients taking statin and aspirin in the Q2 group (*p* < 0.05 for all).

### 3.2. Increased RAGE Expression in EAT Is Associated with Greater Echocardiographic EAT Thickness and Higher Adiposity Indices

**Figure 1:**
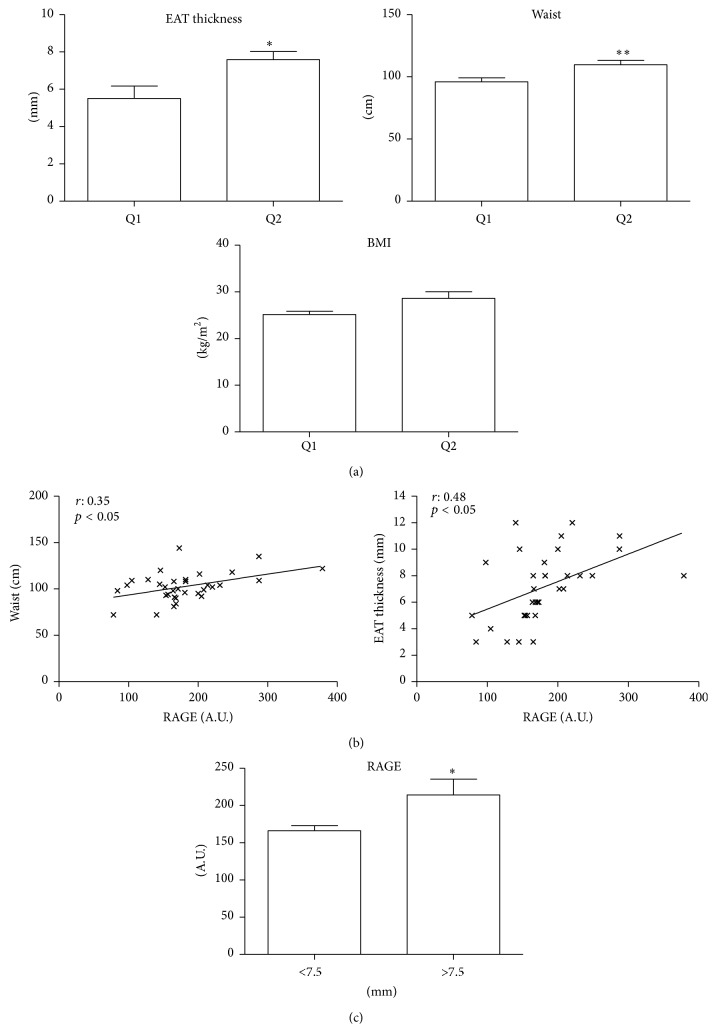
Relationship between RAGE expression in EAT, EAT thickness, and anthropometric indices in CAD patients. (a) CAD patients were stratified into two groups (Q1 and Q2) on the basis of the median RAGE expression in EAT, and EAT thickness, waist circumference, and BMI were compared in the two groups. (b) Spearman correlation analysis between mRNA RAGE level in EAT and waist circumference and EAT thickness. (c) CAD patients were stratified into two groups (Q1 and Q2) on the basis of the median EAT thickness (7.5 mm) and the levels of RAGE expression were compared in the two groups. A.U.: arbitrary unit. Data are expressed as mean ± SD; ^*∗*^ *p* < 0.05, ^*∗∗*^ *p* < 0.01.

Echocardiographic EAT thickness and WC, a marker of visceral fat distribution, were higher in group Q2 than Q1 (*p* < 0.05 and *p* < 0.01, resp.) (Figure 1(a) and Table 1). Positive correlations were seen between RAGE-EAT thickness (*r* = 0.48, *p* < 0.05) and RAGE-WC (*r* = 0.35, *p* < 0.05) (Figure 1(b)). The relation between increased RAGE expression and greater EAT thickness was confirmed by classifying patients according to the median EAT thickness (7.5 mm). RAGE expression in the upper group (EAT thickness > 7.5 mm) was about 1.3 times higher (*p* < 0.05) than in the lower group (EAT thickness < 7.5 mm) (Figure 1(c)).

### 3.3. Increased RAGE Expression in EAT Is Associated with Lower GLUT4 and Adiponectin Expression and a Higher LAP Index

**Figure 2:**
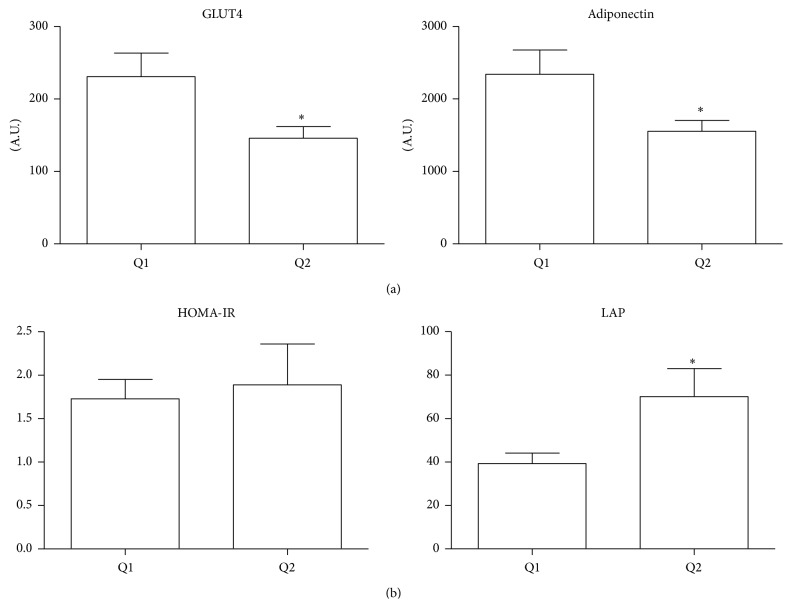
Relationship between RAGE expression in EAT and indices of insulin sensitivity. CAD patients were stratified into two groups (Q1 and Q2) on the basis of the median RAGE expression in EAT. (a) Gene expression of GLUT4 and adiponectin in EAT was compared in the two groups. (b) HOMA-IR and LAP were compared in the two groups. Data are expressed as mean ± SD; ^*∗*^ *p* < 0.05.

Patients in the upper group of RAGE expression in EAT (Q2) had about 1.6 times lower levels of both the insulin-sensitizing adipokine adiponectin and the insulin-responsive glucose transporter 4 (GLUT4) than in the Q1 group (*p* < 0.05 for both) (Figure 2). There was an inverse correlation between RAGE-adiponectin (*r* = −0.40, *p* < 0.05) and RAGE-GLUT4 (*r* = −0.60, *p* < 0.001).

Examining the relation between RAGE expression in EAT and LAP and HOMA-IR, two parameters of insulin resistance, only LAP was higher in Q2 than in Q1 (Figure 2).

### 3.4. Increased RAGE Expression in EAT Is Associated with Lower Expression of the Antioxidant Glyoxalase 1 System and Higher HMGB1, TLR-4, and MyD88

**Figure 3:**
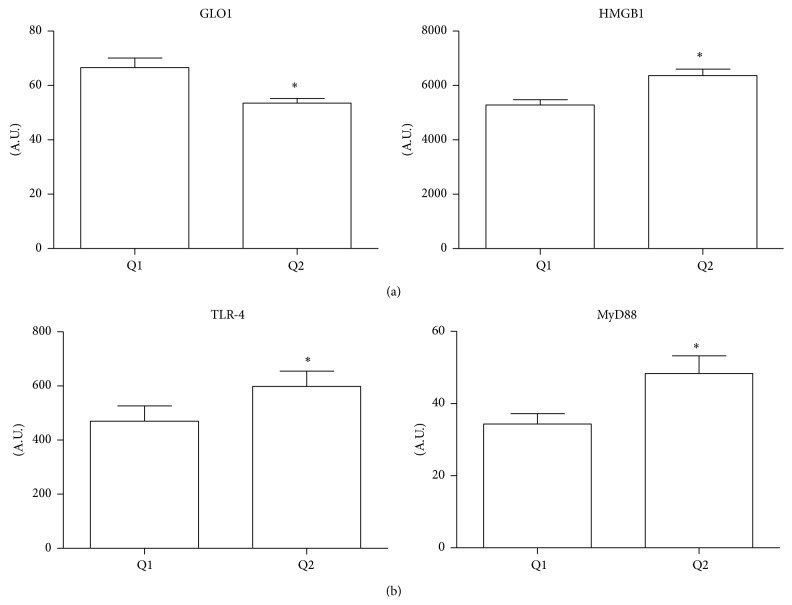
Relationship between RAGE expression in EAT and EAT inflammation. CAD patients were stratified into two groups (Q1 and Q2) on the basis of the median RAGE expression in EAT. (a) Gene expression of GLO1 and HMGB1 in EAT was compared in the two groups; (b) TLR-4 and MyD88 levels were compared in the two groups. A.U.: arbitrary unit. Data are expressed as mean ± SD; ^*∗*^ *p* < 0.05.

Patients in the Q2 group of RAGE expression in EAT had about a 1.3 times lower level of glyoxalase 1 (GLO1), a system playing a critical role in the prevention of glycation reactions mediated by methylglyoxal, glyoxal, and other RAGE ligands, and about 1.2 times the level of the endogenous RAGE ligand HMGB1 compared to group Q1 (*p* < 0.01 for both) (Figure 3(a)). Both TLR-4 and MyD88, important in the activation of the innate immune system, were higher in group Q2 (about 1.4 times, *p* < 0.05 for both) (Figure 3(b)).

Correlation analyses indicated an inverse correlation between RAGE and GLO1 (*r* = −0.65, *p* < 0.0001) and a positive association between RAGE-TLR-4 (*r* = 0.51, *p* < 0.001) and RAGE-MyD88 (*r* = 0.48, *p* < 0.01).

## 4. Discussion

To the best of our knowledge this is the first human study exploring the existence of an association between the expression of RAGE in EAT, EAT metabolic dysfunctions, and adiposity in CAD patients. The findings indicate that EAT thickness as well as local tissue inflammation and insulin sensitivity seems related to local expression of RAGE.

Previous* in vitro* and animal studies suggested that RAGE could be involved in the progression of obesity, with a direct role in promoting adipocyte hypertrophy [7]. RAGE −/− mice at 20 weeks of age had lower weight and lower epididymal adipose tissue weight and adipocyte size than wild type mice [7]. The observation that adenoviral RAGE overexpression in 3T3-L1 adipocytes markedly induced a hypertrophic phenotype, which was suppressed by RAGE silencing, also supports a role for RAGE in promoting adipocyte hypertrophy [7].

Our data seem to confirm that RAGE is involved in adiposity, mainly visceral, in humans too. In fact, with increasing local expression of RAGE, we observed increases in the thickness of EAT, an acknowledged visceral fat, and in WC, which serves as a marker of visceral fat accumulation. Whether this RAGE-related EAT expansion involved adipocyte hypertrophy needs to be explored further. In fact, we noted a hypertrophic state associated with the increased RAGE expression (data not shown), but since the number of patients with enough tissue for this analysis was limited, these results can only be considered preliminary.

In this study we did not directly explore the mechanisms promoting RAGE upregulation, but on the basis of previous data also from our group it would appear that both the increased inflammatory state described in EAT in CAD patients and the greater local accumulation of AGE products in expanding adipose tissue may promote this [9, 12, 15–17]. The increase in the local production of damaging agents and reduced protection against them is also borne out by the marked reduction in GLO1, the major detoxification enzyme that protects against AGE [18], and the higher expression of HMGB1, an endogenous mediator of inflammation able to bind RAGE, promoting its expression and amplifying the inflammatory response also through activation of toll-like receptor (TLR)/MyD88 pathways. It has recently been suggested that RAGE not only shares several common ligands with the TLRs, such as HMGB1, but may also interact with MyD88, an important intracellular adaptor protein used by these receptors [19]. There is increasing evidence, therefore, of their potential synergism in amplifying inflammatory responses and our findings too suggest a link between TLR-4/MyD88 and RAGE hyperexpression.

Our data also confirm that some important metabolic dysfunctions of EAT in patients with CAD may be related to RAGE overexpression. Monden et al. [7] indicated that RAGE overexpression reduced the genes involved in insulin sensitivity, such as GLUT4 and adiponectin, and attenuated insulin function. We too saw lower levels of both GLUT4 and adiponectin, with increased RAGE expression in EAT. This suggests a potential impairment of local insulin signaling. We examined insulin sensitivity in our CAD patients using the HOMA-IR, a marker of insulin resistance mainly in the liver, and LAP, a continuous variable based on WC and triglyceride concentration, two parameters which reflect tissue lipid accumulation and denote visceral adiposity [20, 21]. Our observation that RAGE expression in EAT was mainly related to LAP reinforced the idea of a strong correlation between local RAGE overexpression, fat accumulation, and impaired insulin sensitivity.

Our study has some limitations. The first one is the lack of data on protein expression. Since isolation of EAT during surgery is a delicate and difficult procedure and the amount of tissue isolated is often poor and not enough to perform both gene and protein expression analyses, in this study we first decided to carry out a gene expression study. The lack of quantification of local AGE as well as the evaluation of other molecules, which may promote RAGE upregulation, may also represent a second important limit. The third limitation is that we included only males, so presently we cannot check for possible gender-related differences. Only through new patient enrollment, of both sexes, we will be able to perform protein expression quantification to study which specific pathways/molecules drive RAGE expression in EAT as well as clarify the existence of potential gender-related differences. Finally, the comparison between EAT and other kinds of fat depots, not performed in this study, could be helpful to reinforce our data on the role of RAGE in linking EAT metabolic dysfunction and CAD.

## 5. Conclusions

In conclusion this study's findings suggest the potential involvement of RAGE in promoting EAT dysfunction in CAD patients. Whether RAGE could also be a potential target to reduce EAT-induced cardiovascular and other complications needs to be explored further.

## Figures and Tables

**Table 1:** Demographic, anthropometric, and biochemical characteristics of coronary artery disease patients included in the study before and after classification according to the median value of RAGE expression at EAT level (Q1 and Q2 groups).

	Median	25th–75th percentiles	Range	Q1 (mean ± SD)	Q2 (mean ± SD)	*p*
Age (years)	68.00	57.50–71.70	50.00–86.00	68.18 ± 2.99	66.19 ± 2.09	0.67
Weight (Kg)	75.00	65.75–81.50	52.00–135.00	72.88 ± 2.84	84.17 ± 6.12	0.33
BMI (kg/m^2^)	26.20	23.76–27.90	19.12–41.80	25.14 ± 0.71	28.62 ± 1.44	0.08
Waist (cm)	102.00	93.50–109.05	72.00–144.00	95.94 ± 3.25	109.60 ± 3.61	**<0.01**
EAT thickness (mm)	7.50	5.78–8.00	3.00–10.00	5.50 ± 0.67	7.58 ± 0.44	**<0.05**

Fasting glucose (mg/dl)	82.50	77.50–104.50	64.00–177.00	99.24 ± 7.83	91.53 ± 6.59	0.79
Fasting insulin (*μ*U/ml)	7.11	4.17–10.93	3.19–45.06	6.97 ± 0.71	9.07 ± 1.90	0.53
HbA1C (%)	4.68	3.53–5.52	2.79–7.10	4.76 ± 0.36	4.58 ± 0.30	0.71
Total cholesterol (mg/dl)	153.00	138.00–180.30	88.00–261.00	169.80 ± 10.20	146.40 ± 6.81	0.08
HDL cholesterol (mg/dl)	44.00	34.25–49.25	23.00–69.00	44.13 ± 2.50	41.73 ± 3.53	0.58
LDL cholesterol (mg/dl)	83.60	71.50–109.30	17.60–192.00	104.40 ± 9.89	82.57 ± 4.45	0.07
Triglycerides (mg/dl)	107.00	88.50–143.00	64.00–244.00	114.60 ± 9.43	130.10 ± 15.57	0.40
CRP (mg/dl)	0.20	0.10–0.95	0.00–7.90	0.76 ± 0.37	1.23 ± 0.54	0.44
Systolic blood pressure (mmHg)	130.00	120.00–140.00	110.00–150.00	129.10 ± 2.51	131.30 ± 3.26	0.61
Diastolic blood pressure (mmHg)	70.00	70.00–80.00	60.00–80.00	74.55 ± 1.58	83.00 ± 1.49	0.10

	*n*	%		Q1 (*n*, %)	Q2 (*n*, %)	*p*

Smokers	14	42.42		6, 42.85	8, 57.15	0.73
Diabetes mellitus	9	27.27		4, 44.44	5, 55.56	0.70
Hypertension	24	72.73		11, 45.83	13, 54.17	0.44
Dyslipidemia	20	60.61		7, 35.00	13, 65.00	**<0.05**

Aspirin	19	57.58		6, 31.58	13, 68.42	**<0.05**
Antidiabetics	6	18.18		3, 50.00	3, 50.00	1
ACEI/ARB	24	72.73		10, 41.67	14, 58.33	0.12
*β*-Blockers	16	48.48		7, 43.75	9, 56.25	0.49
Calcium channel blockers	6	18.18		3, 50.00	3, 50.00	1
Statins	22	66.67		8, 36.36	14, 63.64	**<0.05**

ACEI: angiotensinogen-converting enzyme inhibitor; ARB: angiotensin receptor blockade; BMI: body mass index; CRP: C-reactive protein; EAT: epicardial adipose tissue; HbA1C: glycated hemoglobin. Data are expressed as median, 25th–75th percentiles and range or number (*n*), and % or mean ± standard deviation (SD).
